# Investigating Aggregation Using In Situ Electrochemistry and Small-Angle Neutron Scattering

**DOI:** 10.1021/acs.jpcc.2c03210

**Published:** 2022-07-29

**Authors:** Rebecca I. Randle, Ana M. Fuentes-Caparrós, Leide P. Cavalcanti, Ralf Schweins, Dave J. Adams, Emily R. Draper

**Affiliations:** † School of Chemistry, University of Glasgow, Glasgow G12 8QQ, U.K.; ‡ ISIS Neutron and Muon Source User Office, Science and Technology Facilities Council, Rutherford Appleton Laboratory, Harwell Oxford, Didcot OX11 0QX, U.K.; § Large Scale Structures Group, Institut Laue-Langevin, 71 Avenue des Martyrs, CS 20156, F-38042 Grenoble Cedex 9, France

## Abstract

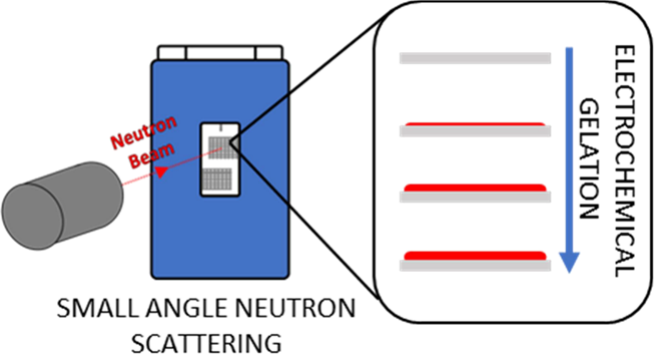

Using small-angle neutron scattering to investigate the aggregation of self-assembling molecules is well established. Some of these molecules are electrochemically useful, for example, in electrochromic devices. Electrochemistry can also be used in some cases to induce aggregation. Here, we describe an approach whereby electrochemistry can be directly carried out on a sample in the neutron beam, allowing us to monitor changes directly in situ. We exemplify with two examples but highlight that there are many other potential opportunities.

## Introduction

The use of small-angle neutron scattering (SANS) is a powerful tool in understanding self-assembling systems over length scales from 0.5 nm to several 100 nm and is used in most scientific fields as the technique is so versatile and useful.^1^ In materials chemistry, SANS can be used to determine the structure of a variety of organic and inorganic materials and also to follow kinetic processes.^2−7^ When SANS is collected over a changing system, this can be described as in situ SANS. For example, in situ SANS can be employed to follow a change in structure over time after an initial stimulus such as a change in temperature or pH, as well as following phenomena such as, but no way limited to, dealloying, ordering, or gelation.^8−13^ For self-assembling systems, small-angle scattering has the advantage over many techniques that drying is not required (that can lead to artifacts and result in unrepresentative characterization of morphology) and provides information on the bulk sample.^14^ Other bulk analysis techniques for these systems include circular dichroism, absorption, nuclear magnetic resonance, and infrared spectroscopy. While these are all powerful techniques, they offer limited information in isolation on specific length scales and the interpretation of these techniques can vary.^15,16^ The setup of the actual experiment may also not be representative of the system; for example, in absorption spectroscopy for highly absorbing systems, samples are often diluted below the minimum gelation concentration to be able to collect the data.^15^ Small-angle scattering (SAS) however offers information over a much wider length scale (from molecule interactions to fiber morphology) without the need for changing the system.^17^

Several self-assembling systems can be triggered electrochemically.^18,19^ Self-assembling small organic molecules are susceptible to changes in aggregation, which in turn can influence properties relevant to electrochemistry such as photoconductivity, charge carrying ability, and efficiency of electrochemical reduction or oxidation.^20−22^ However, SANS measurements can take significant time to produce well resolved data (typically up to 1 h depending on how well the samples scatter).^23^ Species that are not stable for this length of time cannot be reliably measured using SANS; for example, electrochemical changes in some systems require application of a constant potential and so if ex situ application of a current is carried out, the species can degrade or revert to the original state over the course of the SANS experiment.^24^

Performing electrochemistry on a sample in the beam itself has all the advantages of real-time SAS measurements, allowing analysis of a larger variety of length scales than absorbance spectro-electrochemistry (which gives more information about molecular packing rather than overall structure in these types of systems and can be limited by factors such as concentration).^25,26^ The use of in situ electrochemistry combined with SANS and small-angle X-ray scattering (SAXS) (electrochem-SANS and electrochem-SAXS) and with other ex situ techniques has been reported mostly in the evaluation of batteries.^27−29^ A cell in the beam line can be monitored during multiple charge and discharge cycles.^28,30^ In this field, electrochem-SANS has been used to assess pore structure and host nature of electrodes, to give insight into mechanisms and to investigate nanoparticle size.^25,29,31^ Electrochem-SAXS has been performed to monitor platinum nanostructures growing within a lipid template using a small gold substrate working electrode and three-dimensional printed custom holders.^32^ The use of electrochem-SAS to the best of our knowledge has not been applied to organic materials.

SANS and SAXS both have different advantages and disadvantages despite being able to show us the same level of understanding on the different length scales. SAXS does not require contrast between the material and the background (often done by from deuteration of the solvent or the molecule), which can cause issues from isotope effects.^31^ SAXS has greater accessibility as it does not need to be performed at a large facility such as a synchrotron or beamline. SANS does require contrast, but this also provides an opportunity for contrast matching to reveal more about the materials being investigated. For example, materials can be selectively deuterated so that parts of the molecules are “invisible” to the neutrons. This method has been utilized to determine the elongation rate and length of amyloid fibrils^33^ and reveal the packing of molecules in a gel fiber.^2^ Similarly, the background solvent scattering length density can be matched to that of the material, again effectively making a component “invisible”; this is particularly useful in multicomponent systems.^34,35^

Here, we report a simple contained method of performing electrochem-SANS, able to be controlled from outside the beam. We show that we can use this setup to follow different processes using SANS. Only a small volume (2 mL) of solution is required, and the entire setup can be transported in a standard sized rucksack. All components apart from the quartz cuvette are standard and used as purchased. These factors give this method an advantage over other reported electrochem-SANS setups, which required dialysis tubing or large reservoirs of electrolyte.^8,25,36^ The use of the software is relatively straightforward, the cell is easy to remove and replace into the beam, and while delicate, the internal electrodes and quartz can be cleaned quickly between samples.

## Methods

**Figure 1:**
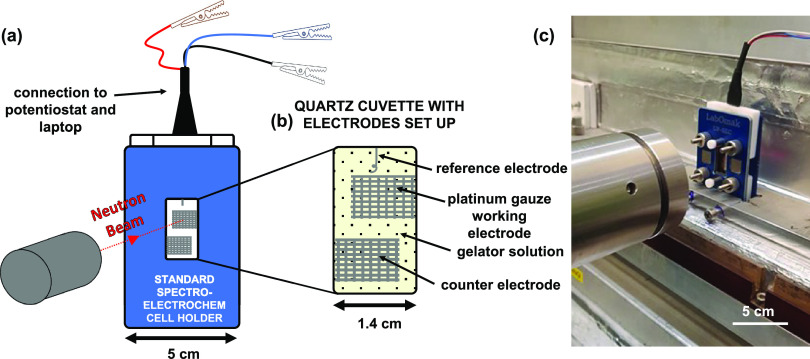
(a) Cartoon of the experimental setup of the spectro-electrochemical cell in the neutron beam; (b) expanded diagram of the cell window where the beam is positioned with a specific example of electrochemical reduction and oxidation of the gelator solution at the working electrode. (c) Photograph of the electrochemical cell in the neutron beam on SANS2D at ISIS.

Full synthetic and experimental procedures are described in the Supporting Information. In situ electrochemistry in the beamline was performed using an adapted LabOmak UF-spectro-electrochemical cell, which normally slots into a spectrophotometer. The cell has a platinum working, counter, and reference electrode (Figures 1a,b and S1). A custom-made 2 mm path length quartz cuvette (Quartz Scientific Glassblowing Ltd., UK) was used to hold these electrodes and solution in place and replaces the standard calcium fluoride windows. The 7 × 10 mm (width × height) neutron beam was then focused onto the working electrode so we could monitor the changes at its surface where the electrochemical processes were taking place, which caused structural changes. The position of the beam was set manually with the aid of a custom-made plate (Figures 1c and S2–4) to hold the spectro-electrochemical cell.

Due to the simplicity of the setup, the electrochemical cell could be easily replaced with a standard sample changer in the night, for example, without changing the beam conditions, enabling users to maximize the beamtime. A PalmsSens4 potentiostat (Alvatek Ltd.) was used to apply the required currents. Extension wires were used to connect the potentiostat to a laptop located outside of the beamline “hutch”, which could control the measurements via PSTrace software (Version 7.2),^37^ but also could be connected to the laptop with a Bluetooth connection.

### Kinetic Experiments

**Figure 2:**
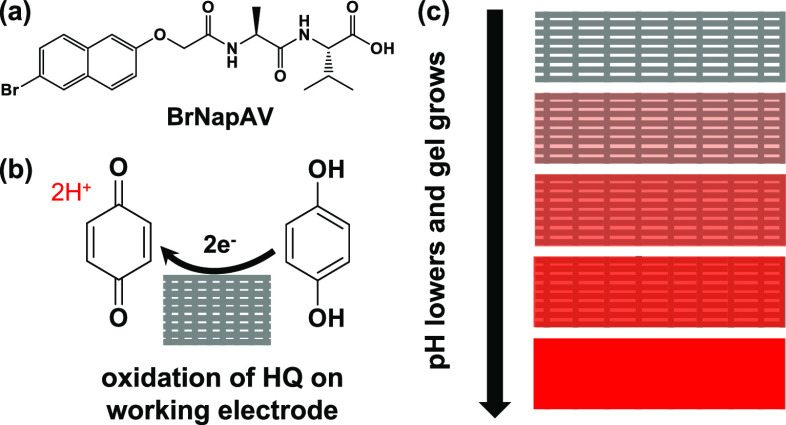
(a) Chemical structure of BrNapAV; (b) oxidation of HQ on the working electrode; (c) lowering the pH via HQ oxidation on the working electrode resulting in localized gel growth. The gel is represented by a red color for clarity.

Solutions of BrNapAV (Figure 2a) were prepared at 5 mg/mL of gelator and 1 M equivalent of NaOD (0.1 M, aq.) incorporating hydroquinone (HQ) and sodium chloride (0.065 and 0.1 M, respectively). The pD was adjusted to 8 after all the solid was dissolved by stirring with a magnetic stirrer overnight. Solutions of BrNapAV were gelled using the oxidation of HQ. A potential of 30 μA was applied for 1 h, and the scattering at 8 m was collected at the mid Q range (7.8 × 10^–3^ up to 1.3 × 10^–1^ A^–1^) every 5 min. After 60 min, the current was stopped, and the scattering of the final gel was collected at low, mid, and high Q ranges. Electrochemical gelation experiments were performed using a D11 instrument (Institut Laue Langevin, Grenoble, France). A neutron beam, with a fixed wavelength of 6 Å and divergence of Δλ/λ = 9%, allowed measurements over a large range in a *Q* [*Q* = 4πsin(θ/2)/λ] range of 0.001 to 0.3 Å^–1^, by using three sample-detector distances of 1.5, 8, and 39 m. For the kinetics, only 8 m was used and a measurement collected every 5 min.

### In Situ Reduction of NDI-GF

Solutions were prepared at a concentration of 10 mg/mL of NDI-GF (Figure 4). NDI-GF solids were dissolved in 2 molar equivalents of aqueous NaOD (0.1 M), and the remaining volume was made up with a pD 6 buffer. To reduce the sample, a potential of −0.7 V was applied while the measurement was being performed. To then oxidize the sample, a potential of +0.6 V was applied for 10–15 min and another measurement was taken. These potentials were taken from cyclic voltammograms measured using the same cell (Tables S1 and S2). Electrochemical reduction in the beamline was performed on SANS2D (STFC ISIS Pulsed Neutron Source, Oxfordshire, UK). The beamline setup was a 4 m sample-to-detector distance, a beam size of 8 mm, and a typical *Q*-range [*Q* = 4πsin(θ/2)/λ, where *q* is the scattering angle] from 0.004 to 0.7 Å^–1^ set by time-of-flight mode with incident wavelengths (λ) from 1.75 to 16.5 Å.

### Ex Situ Reduction of NDI-GF

Solutions were prepared as previously described using a deuterated solvent and base. All solutions were prepared with deuterated buffers. pD was adjusted using 0.1 M NaOD and DCl. Samples were transferred to an fluorine-doped tin oxide (FTO) window cell and reduced and oxidized using −2.5 and 0.5 V, respectively (Figure S5). These values were taken from cyclic voltammograms (Table S2). The measurements in cuvettes were performed using a SANS2D instrument (STFC ISIS Pulsed Neutron Source, Oxfordshire, UK). A multiple-slot sample changer with a controlled temperature of 25 °C was used. The beamline setup was a 4 m sample-to-detector distance, a beam size of 8 mm, and a typical *Q*-range [*Q* = 4πsin(θ/2)/λ, where *q* is the scattering angle] from 0.004 to 0.7 Å^–1^ set by time-of-flight mode with incident wavelengths (λ) from 1.75 to 16.5 Å. Samples were placed in 2 mm quartz cuvettes and measured for ∼60 min.

All scattering Isis data were normalized for the sample transmission and background corrected (0.1 M buffers made in D_2_O), and data reduction was performed using a Mantid framework installed inside the ISIS virtual machines, IDAaaS. All the scattering data were then fitted in the SasView software (version 4.2.2).^38^

## Results and Discussion

As a first exemplar of the success of this approach, we used the in situ electrochemical gelation. Here, gels can be formed using pH-triggered low molecular weight gelators (LMWGs) by inducing a pH change at an electrode surface.^18,39,40^ This is achieved by the electrochemical oxidation of HQ to benzoquinone, which results in the release of protons. We have described this approach elsewhere and shown that the volume of the gel can be controlled by the potential applied and the time of application.^39^ We note that electrochemical and other processes can be different when using D_2_O compared to H_2_O;^41,42^ however, for these described systems, we observe little or no difference in the gelation or electrochemistry. Therefore, the effect of D_2_O on systems should be explored before in situ experiments are carried out.

**Figure 3:**
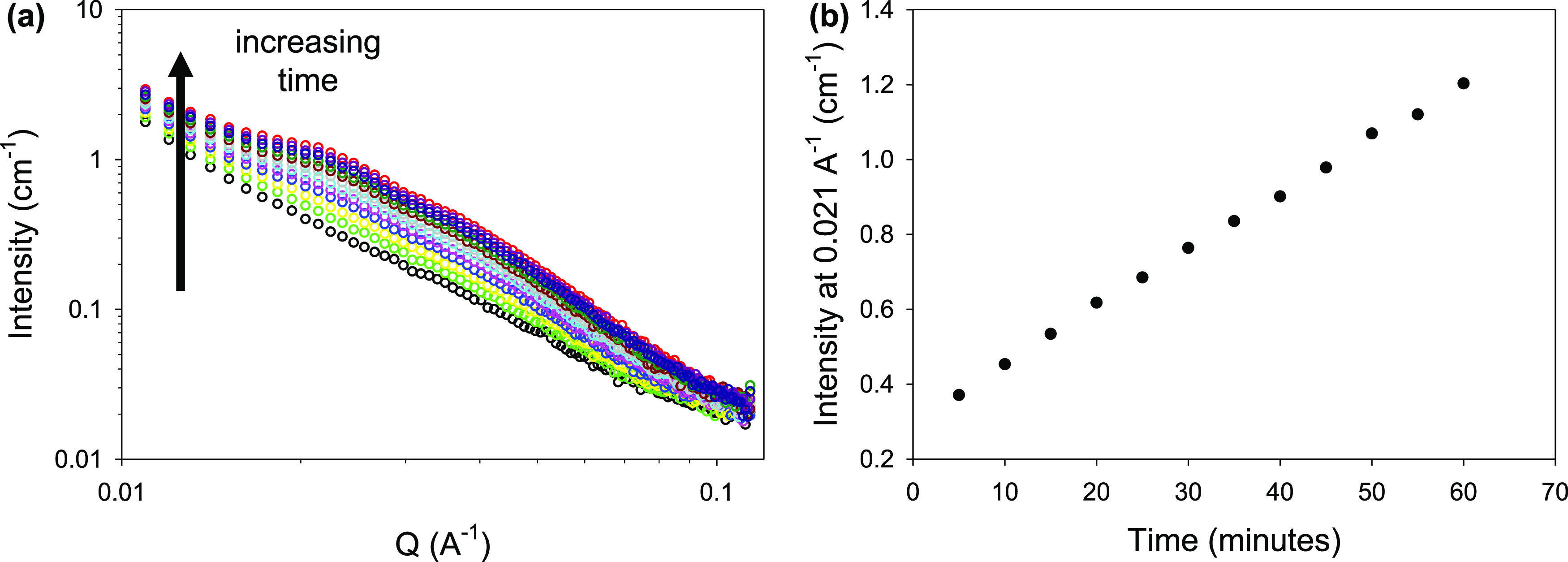
(a) In situ SANS during the electrochemical gelation of BrNapAV using 30 μA. Scattering was collected every 5 min for an hour. (b) Intensity of the scattering at 0.021 Å^–1^ (where a feature in the scattering grows in) during gelation, showing a linear growth of the gel.

A well-established LMWG was examined, BrNapAV (Figure 2a).^43^ A free-flowing solution is formed at pH 8 in the presence of sodium chloride and HQ; at this pH, the terminal carboxylic acid is deprotonated. At this point, the solutions are weakly scattering (Figure S7), indicative of the absence of significant self-assembly. On application of a potential of 30 μA, oxidation of HQ begins, and a gel starts to form on the electrode surface (Figure 2b,c). The intensity of the scattering increases with time as the gel forms (Figure 3). After 60 min, the SANS data can be best fit to the elliptical cylinder model combined with a power law, similar to data from pH-triggered gels formed in the bulk (Figure S8 and Table S3).^44^ This shows that the structures underpinning the gels are the same in the electrochemically triggered gels as those in gels formed by more traditional pH triggers, which has not previously been shown. The gel grows linearly (Figure 3b), which agrees with other studies looking at the electrochemical gelation with in situ surface plasmon resonance spectroscopy.^39,40^

**Figure 4:**
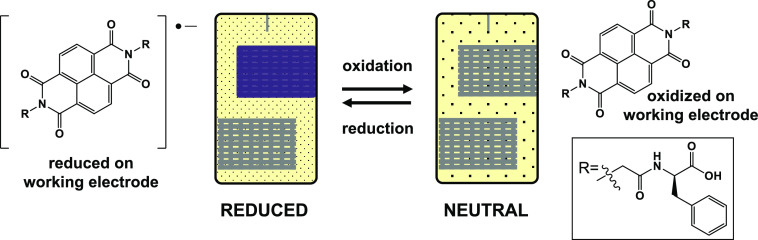
Cartoon showing the electrochemical reduction and oxidation on the working electrode surface of NDI-GF. Reduction results in a color change (shown here as purple for clarity) due to the radical anion being generated, which can be oxidized back to the neutral state.

As a second exemplar, we have used this in situ approach for the pH dependent aggregation of water-soluble electrochromic naphthalene diimide (NDI) species.^45^ The type of aggregation greatly impacts the efficiency of electrochemical oxidation and reduction (processes essential to a chromic device).^46^ We previously reported that these systems (Figure 4) have an “ideal” pH at which the electrochemical processes of reduction and oxidation were enhanced due to favorable aggregation.^46^ Here, we are looking at the electrochemical reduction of NDI-GF (Figure 4), which changes color from a colorless solution when neutral to a dark black/brown color upon electrochemical reduction to the radical anionic species (Figure S9). Organics for electrochromics often come under the scrutiny of degradation due to this radical anion formation and so stability and longevity is key analysis, which is needed here. This is where SANS can tell us how the aggregates behave after redox cycling, indicating their stability.

**Figure 5:**
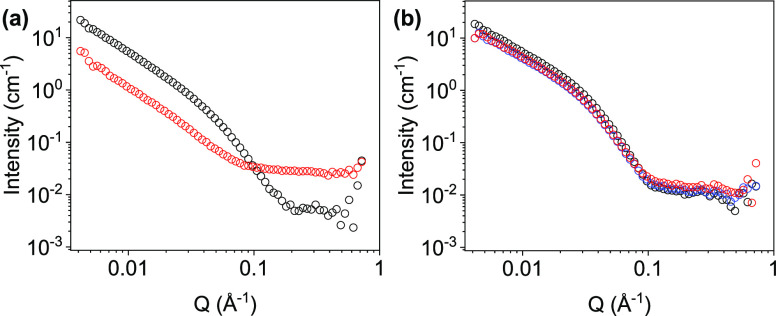
(a) Ex situ SANS from NDI-GF before reduction (black data) and after reduction (red data). (b) In situ electrochem-SANS from NDI-GF before reduction (black data) during reduction (red data) and after electrochemical oxidation (blue data).

To exemplify the need for the in situ measurements, ex situ measurements were carried out. Solutions were reduced and oxidized in a FTO window cell (Figure S9). Samples were then extracted from the cell and collected in a vial before being transferred to a quartz cuvette (Figures S5 and S6 and Tables S1 and S2). The data before and after the cycle are best fitted to a flexible elliptical cylinder combined with a power law model (Figures S10 and S11 and Tables S4 and S5). There was a large difference in the SANS data collected before and after the reduction and oxidation cycle with a reduction in the Kuhn length from 60 to 45 Å and radii from 19 to 7 Å before and after the cycle, respectively (Figure 5a). The axis ratio increases significantly from 1.8 to 6, and the scattering intensity also significantly decreases at low *Q*. It was unclear whether the sample had been degraded during the redox processes or whether it was a consequence of the loading, aging, the uptake of water into D_2_O, or transporting of the sample prior to the SANS measurement.

Using the electrochem-SANS setup removes any ambiguity in the previous data. It was seen that SANS data for the sample prior to a reduction and oxidation cycle are comparable to those for the solution prepared ex situ, illustrating that the setup itself does not impact the scattering (Figure S12). There are some small discrepancies, which is expected from the differences in the setup, but the data can be fitted to a very similar model (Table S6). However, unlike the data from the ex situ experiment, the SANS data collected after an electrochemical cycle in the spectroelectrochemical cell are comparable to those from the initial neutral state (Figures 5b and S13–15 and Table S6). The process of both reduction and oxidation has minimal influence upon the fit parameters, with the Kuhn length and radius differing only by around 2 Å, which is within error. These data are completely different to the previous result collected ex situ and show that the sample is not being affected by the redox process, rather by the transferring and loading of samples. This changes the outcome of this material, as it would have been disregarded for the application of electrochromics, due to degradation. However, with the in situ method, it demonstrates stability to these processes and so is suitable to be taken for further investigations.

## Conclusions

The use of in situ electrochem-SANS has been demonstrated for the first time on organic self-assembled materials. Furthermore, it is an easily accessible, affordable, and usable cell setup. It was used to monitor the scattering from the controlled growth of an electrochemically grown gel in situ, allowing us to see that the gel grew linearly with time of applied current. We also looked at the effect of generating the radical anion electrochemically in an electrochromic system on the aggregates in solution, allowing us to assess any changes occurring to the supramolecular structures before, during, and after this process, something we have been unable to do ex situ. This is an invaluable insight into these types of systems. This allows us to properly characterize any change or degradation in our samples during electrochemical reduction of the material. We can envisage this technique being used on other electrochemically active gel systems^47^ or for applications such as electrochemical sensing.^48^ However, there are opportunities to use this method for any systems where structural change occurs on the working electrode.

## Supporting Information Available

Details of synthesis, experimental setup, protocols, and supplementary data; SANS datasets available through their associated DOIs referenced in the text (PDF)

## Abbreviations

SANS: small-angle neutron scattering
FTO: fluorine-doped tin oxide
HQ: hydroquinone
NDI: naphthalene diimide
LMWG: low molecular weight gelator
SAS: small-angle scattering
SAXS: small-angle X-ray scattering
SLD: scattering length density
