# Supplementary material for: Investigating Aggregation Using In Situ Electrochemistry and Small-Angle Neutron Scattering
Source: J Phys Chem C Nanomater Interfaces. 2022 Jul 29;126(31):13427–32. doi: 10.1021/acs.jpcc.2c03210 (PMC9376955; doi:10.1021/acs.jpcc.2c03210)
Supplement: Supplementary file 1 — jp2c03210_si_001.pdf [file jp2c03210_si_001.pdf]

Investigating Aggregation using *in situ* Electrochemistry and Small Angle Neutron Scattering

Rebecca I. Randle,[a] Ana M. Fuentes-Caparrós,[a] Leide P. Cavalcanti,[b] Ralf Schweins,[c] Dave J. Adams[a] and Emily R. Draper*[a]

[a] School of Chemistry, Joseph Black Building, University of Glasgow, Glasgow, G12 8QQ, UK

[b] ISIS Neutron and Muon Source User Office, Science and Technology Facilities Council, Rutherford Appleton Laboratory, Harwell Oxford, Didcot, OX11 0QX, UK

[C] Large Scale Structures Group, Institut Laue-Langevin, 71 Avenue des Martyrs, CS 20156, F-38042 Grenoble, CEDEX 9, France

*Corresponding author email: Emily.Draper@glasgow.ac.uk

SUPPORTING INFORMATION

Contents

1. Synthetic procedures S2
2. Equipment and protocols S2
3. Supplementary figures S9
4. References S16

1. Synthetic Procedures

All chemicals and solvent were purchased from Merch Life Sciences or Alfa Aesar and used as received with the exception of naphthalenetetracarboxylic dianhydride (NTCDA). NTCDA was purchased from Fluorochem, Alfa Aesar and Merch Life Sciences but impurities were found by NMR in all supplier batches. Following this, all NTCDA was purchased from Fluorochem and purified. The synthetic procedures, purification and characterisation have been reported previously.[1, 2, 5]

2. Equipment and protocols

PREPARATION OF SOLUTIONS -

BrNapAV for electrochemical gelation: Solutions were prepared at 5 mg/mL of gelator and 1 M equivalent of NaOD (0.1 M, aq.) incorporating hydroquinone (HQ) and sodium chloride (0.065 M and 0.1 M, respectively). The pD was adjusted to 8 after all the solid has dissolved after stirring with a magnetic stirrer overnight.

NDI-GF for electrochemical reduction: Solutions were prepared at concentrations 10 mg/mL of NDI-GF. NDI solids were dissolved in 2 molar equivalents of aqueous NaOD (0.1 M) and the remaining volume made up with a pD 6 buffer. Solutions were stirred overnight until all solid had dissolved. Buffered solutions were prepared using 0.1 M sodium acetate, adjusted to pH 6. The pH of solutions was adjusted using 0.1M DCl and NaOD and pH of buffers adjusted with 1 M DCl and NaOD. The sodium acetate buffer was prepared by dissolving 1.64 g of sodium acetate in 200 mL D_2O.

pH MEASUREMENTS - The pD of these solutions was adjusted using a FC200 pH probe (HANNA instruments) with a 6 mm × 10 mm conical tip calibrated with buffers of pH 4, 7 and 10 (HANNA instruments). The stated accuracy of the pD measurements is ±0.1.

ELECTROCHEMISTRY - Cyclic voltammetry, potentiometry and amperometry were carried out using a PalmSens4 potentiostat (Alvatek Ltd). Voltammograms were measured using 0.1 V/s scan rate. Measurements were collected using PSTrace software (Version 7.2).[3]

A LabOmak UF-spectro-electrochemical cell was used conduct all the *in situ* experiments. The cell has a platinum working, counter and reference electrode. A custom-made 2 mm path length quartz cuvette holds these electrodes and solution (2 mL) in place (Figure S1). Due to

the small amount of Pt gauze in the cell, after neutron exposure, the cell was monitored for any induced radioactivity before removing from the setup.

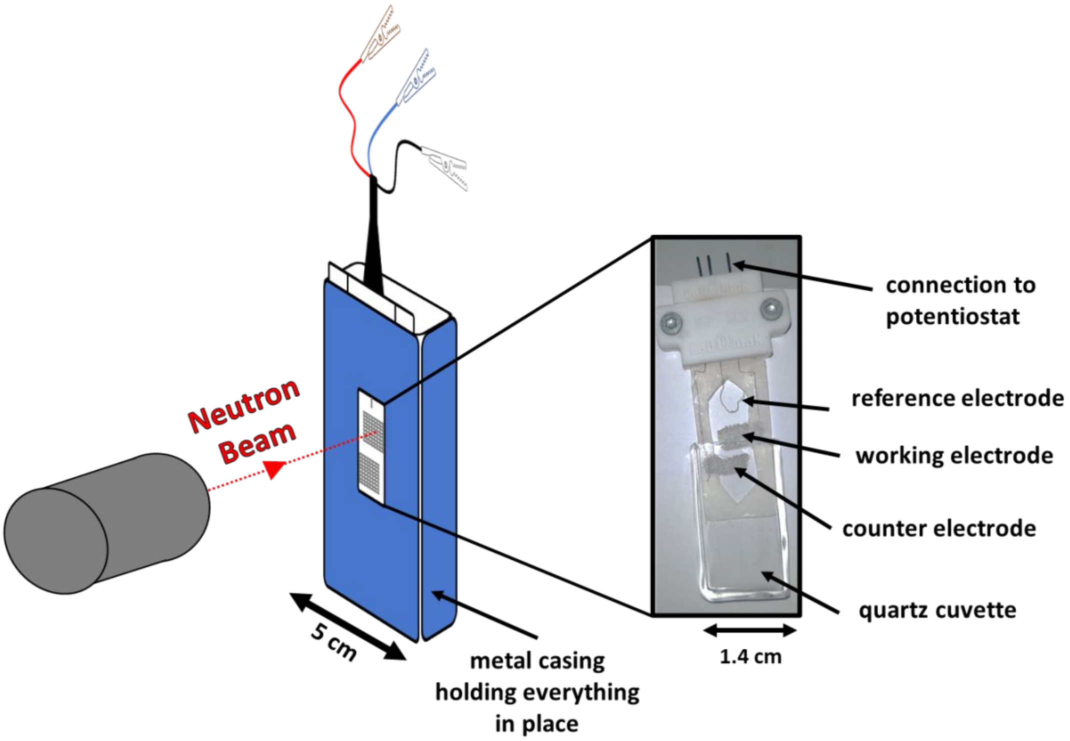

Figure S1. Left – cartoon of the LabOmak UF-spectro-electrochemical cell used in the *in situ* SANS. Right – zoomed in image of the working, reference and counter electrodes in the quartz cuvette.

For the NDI-GF experiments, the electrochemistry was collected using a 2- or 3-electrode setup, depending on whether it was performed in the *in situ* cell, or *ex situ* windows. Reduction and oxidation potentials found from the 2-electrode set up were used for the FTO window cell described in the '*ex situ'* section, whereas potentials found from the 3-electrode set up were used in the beamline experiments.

All data were collected using a 2-electrode setup without reference. Values cannot therefore be stated in reference to a known value. Cyclic voltammograms were carried out using a solution of hydroquinone dissolved in 1 M KCl as the electrolyte. A comparison of the position of the peaks corresponding to the well-defined and recorded quinone-hydroquinone redox reaction in the window cell setup can be made to the same measurement taken in a referenced electrochemical setup (CH Instruments and BASi).

Comparing to a referenced set up, solutions were deposited (5 mL) into a glass vial (CH Instruments) before electrodes were placed into solution. This setup comprised of a glassy carbon working electrode, Pt counter electrode and an Ag/AgCl reference (BASi). Reduction

and oxidation potentials reported must be taken in reference to these more well-defined shifted values, Table S1.

Table S1. Tabulated redox values for hydroquinone in the different electrochemical set ups at a scan rate of 0.1 V/s.

**Redox potentials (V)**	**Cell setup**
-0.15, 0.05	HQ with glassy carbon, Pt counter and Ag/AgCl reference
-2.11, -2.80	HQ in 5 x 5 cm FTO window setup

IN SITU **ELECTROCHEM SANS -** Electrochemical gelation experiments were performed using the D11 instrument (Institut Laue Langevin, Grenoble, France). A neutron beam, with a fixed wavelength of 6 Å and divergence of Δλ/λ = 9%, allowed measurements over a large range in Q [$Q = 4\pi\sin(\theta/2)/\lambda$] range of 0.001 to 0.3 Å-1, by using three sample-detector distances of 1.5 m, 8 m, and 39 m. For the kinetics, only 8 m was used and a measurement collected every 5 minutes. The beamtime allocation 9-11-1966, was on D11 at Institut Laue-Langevin (ILL) Grenoble doi:10.5291/ILL-DATA.9-11-1966.[4]

The data were reduced to 1D scattering curves of intensity vs. Q using the facility provided software. The electronic background was subtracted, the full detector images for all data were normalized and scattering from the empty cell was subtracted. The scattering from D_2O was also measured and subtracted from the data. Most of the data were radially averaged to produce the 1D curves for each detector position. The instrument-independent data were then fitted to the models discussed in the text using the SasView software (Version 4.2.2).[3]

The SLD of the solvent was calculated to be 6.39×10^{-6} Å^{-2}. The SLD of NDI-GF was 3.174 $\times 10^{-6}$ Å^{-2} and for BrNapAV 2.071 $\times 10^{-6}$ Å^{-2}. The best fit was decided to be that which fit well to the data and had the lowest Chi^2 value.

Electrochemical reduction in the beamline was performed on SANS2D (STFC ISIS Pulsed Neutron Source, Oxfordshire, UK). The beamline setup was 4 m sample-to-detector distance, beam size of 8 mm and a typical Q-range [$Q = 4\pi\sin(\theta/2)/\lambda$, where q is the scattering angle] from 0.004 Å^{-1} to 0.7 Å^{-1} set by time-of-flight mode with incident wavelengths (λ) from 1.75 Å to 16.5 Å. STFC beamtime allocation RB2010459 on SANS2D at ISIS Neutron and Muon Source, Dicot doi:10.5286/ISIS.E.RB2010459.[5]

The cell and potentiostat were all set up the sample on each beamline and experiment. The cell was placed in the beamline and connected to a potentiostat, which was controlled from outside the sample area (Figure S2- S4).

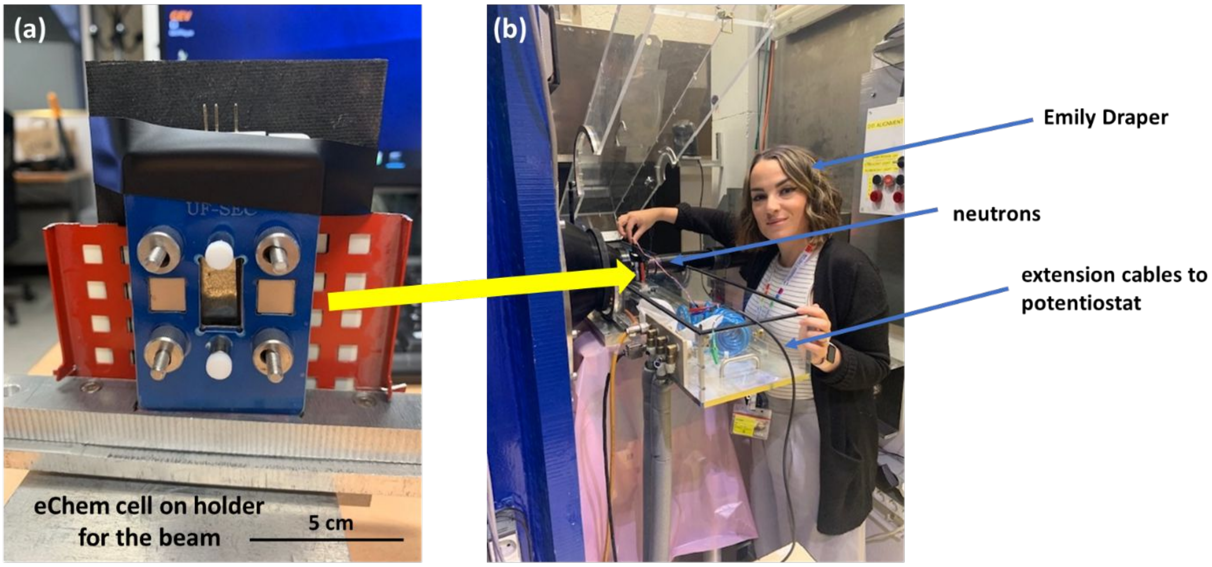

Figure S2. Photographs of (a) the LabOmak UF-spectro-electrochemical cell on the holder used on D11 at ILL, Grenoble (b) the electrochemical cell set up for an experiment on D11.

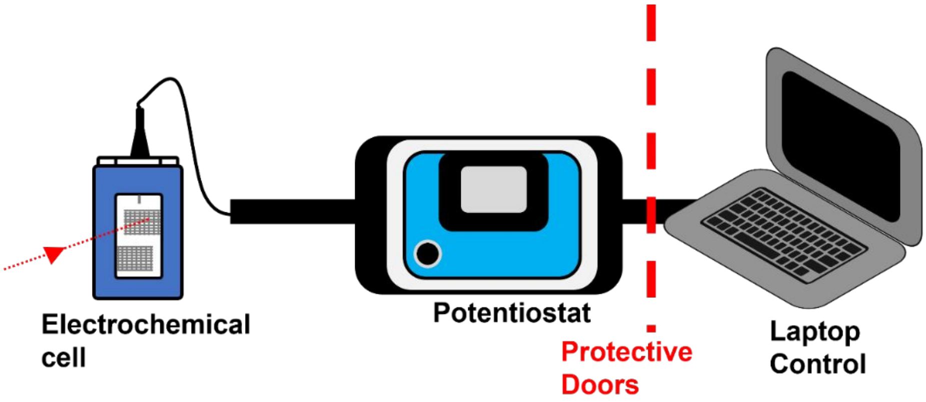

Figure S3. Cartoon of the set up used for the *in situ* electrochem SANS experiments.

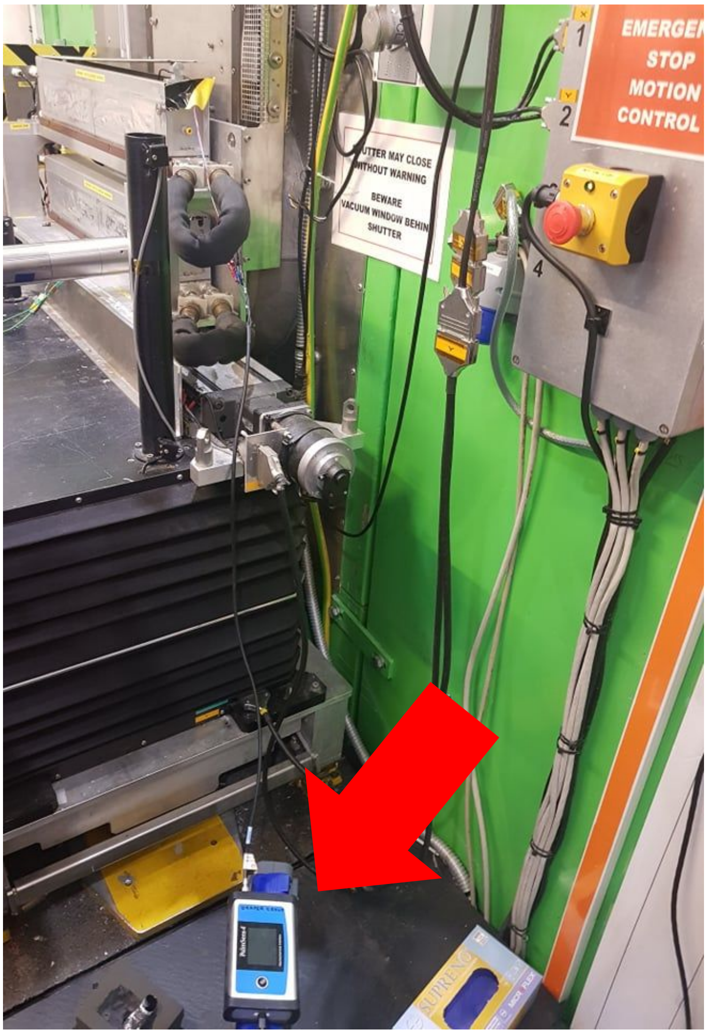

Figure S4. Connection to PalmSense poteniostat to cell on SANS2D at Isis, Didcot (covered by protective foil). Potentiostat is highlighted by red arrow.

***EX SITU* ELECTROCHEM SANS -**The measurements in cuvettes were performed using the SANS2D instrument (STFC ISIS Pulsed Neutron Source, Oxfordshire, UK). A multiple-slot sample changer with controlled temperature of 25°C was used. The beamline setup was 4 m sample-to-detector distance, beam size of 8 mm and a typical Q-range [$Q = 4\pi \sin(\theta/2)/\lambda$, where q is the scattering angle] from 0.004 $Å^{-1}$ to 0.7 $Å^{-1}$ set by time-of-flight mode with incident wavelengths (λ) from 1.75 Å to 16.5 Å. Samples were placed in 2 mm quartz cuvettes and measured for ~60 minutes. The scattering data were normalized for the sample transmission and background corrected 0.1 M buffers made in D_2O and data reduction was performed using Mantid framework installed inside the ISIS virtual machines, IDAaaS. The scattering data were then fitted in the SasView software (version 4.2.2).[6] STFC beamtime allocation RB2010459 on SANS2D at ISIS Neutron and Muon Source, Didcot, UK, Doi:10.5286/ISIS.E.RB2010459.[5]

Solutions were prepared as previously described using deuterated solvent and base. All solutions were prepared with deuterated buffers. pD was adjusted using 0.1M NaOD and DCl.

Samples were transferred to an FTO window cell and reduced and oxidised using -2.5 and 0.5 V respectively (Figure S5). These values were taken from cyclic voltammograms (Table S2).

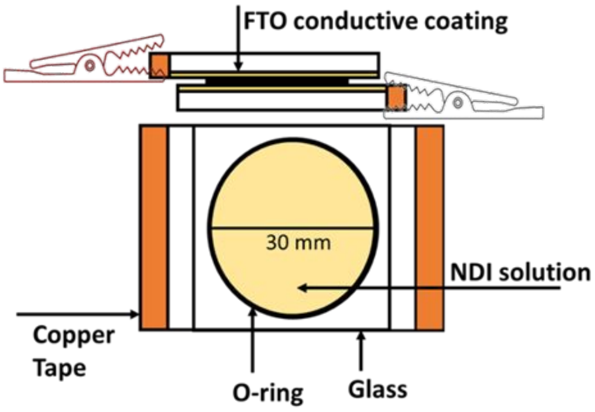

Figure S5. Schematic diagram of the FTO window setup described above.

Table S2. NDI-GF collected at pH 6 at 10 mg/mL within a 5 x 5 cm FTO window setup or platinum working, counter and reference electrodes (*in situ* cell). Scan rate was 0.1 V/s.

	**Reduction Potential(s) (V)**	**Oxidation Potential (V)**
NDI-GF in FTO window	-2.55, -3.24	0.50
NDI-GF *in situ* cell	-0.70	0.60

The window cells used were prepared from fluorine-doped Sn oxide (FTO) coated glass (50 × 50 × 2.2 mm, TEC 7, surface resistivity ~7 Ω/sq, from Sigma Aldrich). Windows were prepared by pipetting solutions of (1-2 mL) onto a piece of glass containing a (0.1 mm) O-ring (30 mm diameter), then securing a second top piece by clamping with bulldog clips. Figure S5, S6 and Figure S9 shows this setup. The glass was sonicated in ethanol at 40°C for 30 minutes prior to assembly and gloves were used throughout to prevent fingerprints on the glass. Pieces of copper tape were added to the edges of the cell to ensure good contact between the cell and the crocodile clips from the PalmSens4 potentiostat. Solutions that were electrochemically used were transferred to and from FTO window cells to carry out electrochemical reduction and oxidation. Solutions were then transferred to a quartz cuvette and measured approximately one week later. Solutions were prepared before arrival on site in advance due to the restrictions in place at the time regarding COVID-19 and postal samples, Figure S6.

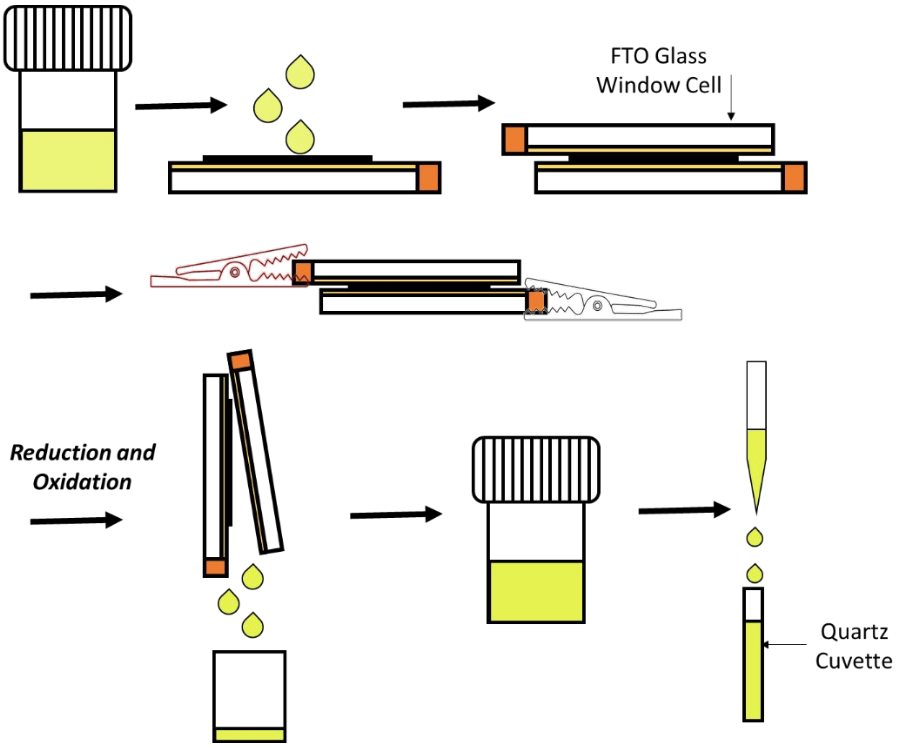

Figure S6. Schematic of how electrochemically used samples were transferred into cuvettes.

3.Supplementary Figures

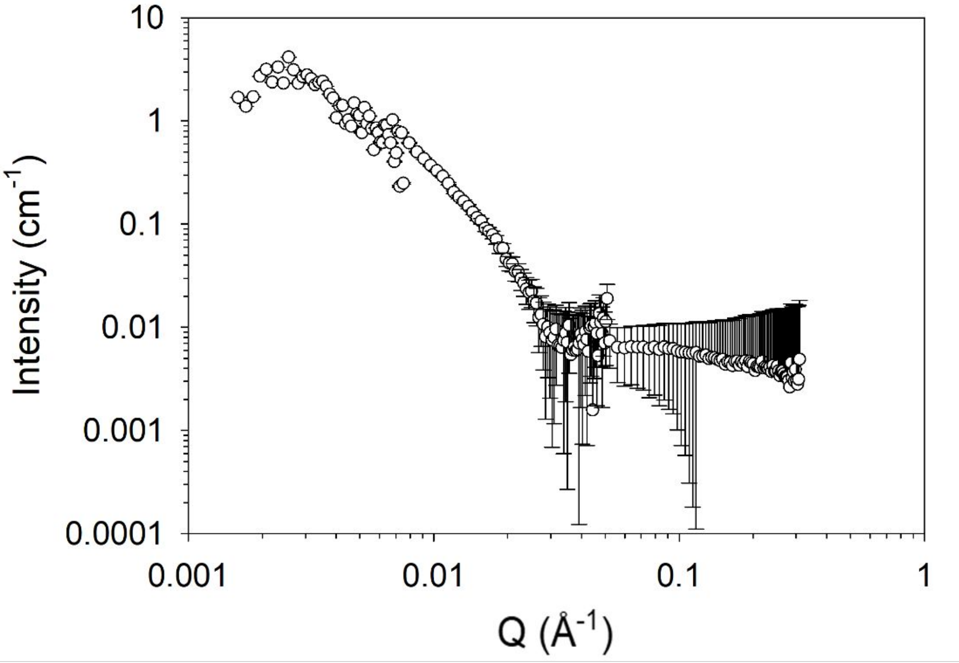

Figure S7. Small angle neutron scattering data for a solution of BrNapAV (t=0, before gelation) showing a weakly scattering material.

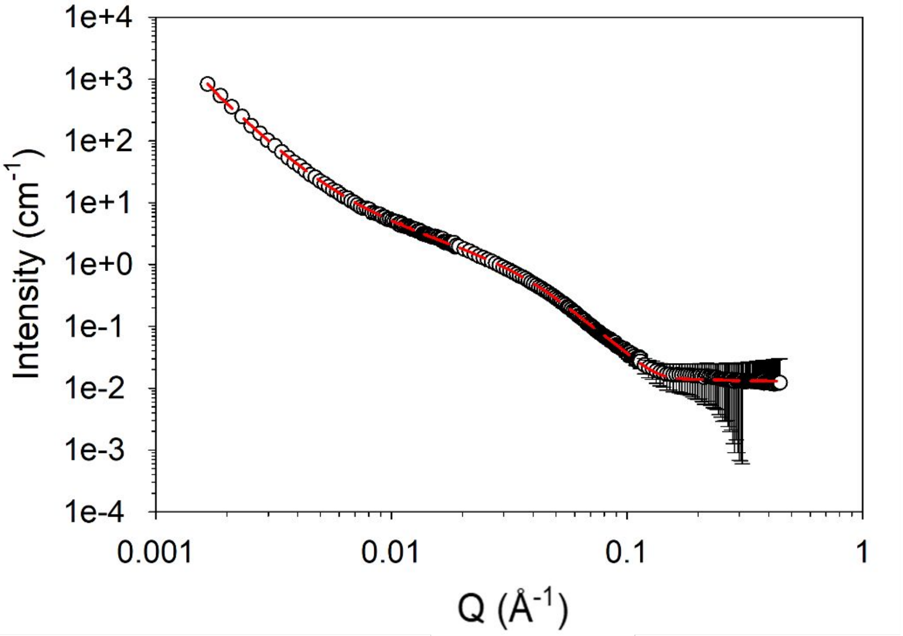

Figure S8. Small angle neutron scattering data for a gel of BrNapAV after 60 minutes of current (black circles) fitted to an elliptical cylinder model combined with power law (red data)

Table S3. Parameters of SANS model fit BrNapAV gel

**BrNapAV gel**	**Elliptical cylinder + power law**
	**Value**
Background (cm^{-1})	0.016 ± 0.000
Scale a	$6.79x10^{-8}$ ± $4.48x10^{-9}$
Power Law	3.62 ± 0.01
Scale b	$1.42x10^{-3}$ ± $2.61x10^{-5}$
Radius (Å)	23.7 ± 0.1
Axis Ratio	2.49 ± 0.01
Length (Å)	2000 ± 458
Chi^2	7.1617

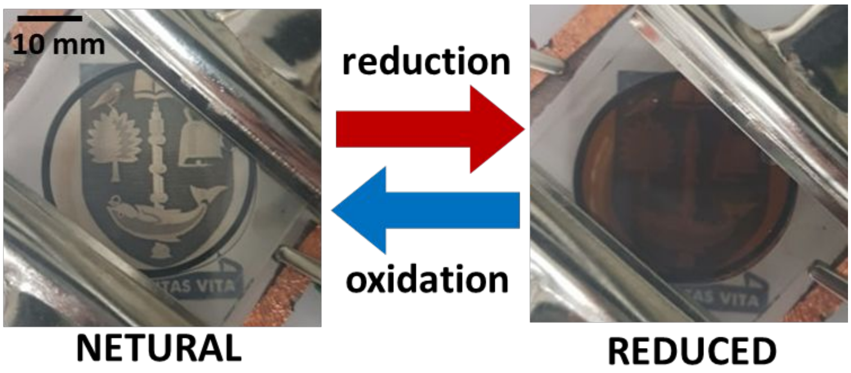

Figure S9. Images of the neutral and reduced colouration of buffered NDI-GF at 10 mg/mL adjusted to pH 9.2. Colouration occurred as a result of application of -2.7 V for 10 seconds.

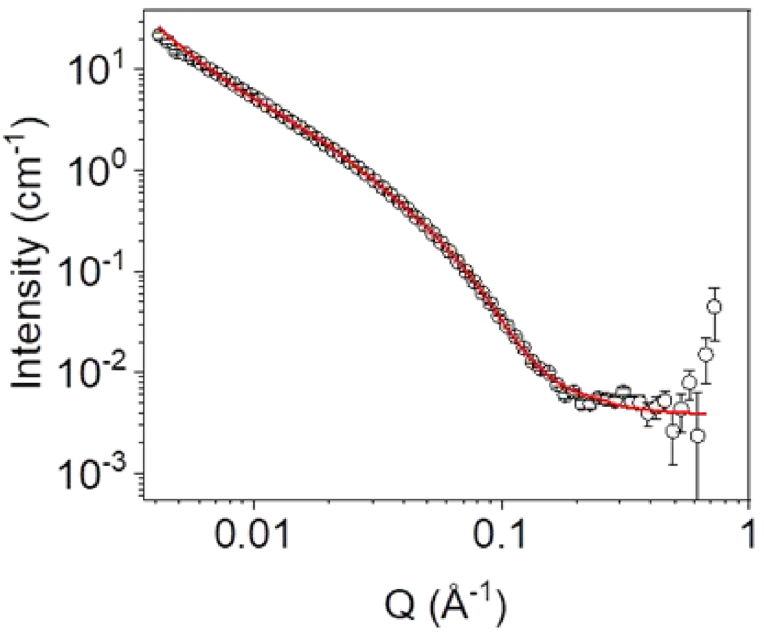

Figure S10. *Ex situ* small angle neutron scattering data for a neutral buffered solution of NDI-GF at pD 6 (black circles) fitted to a flexible elliptical cylinder model combined with power law (red data)

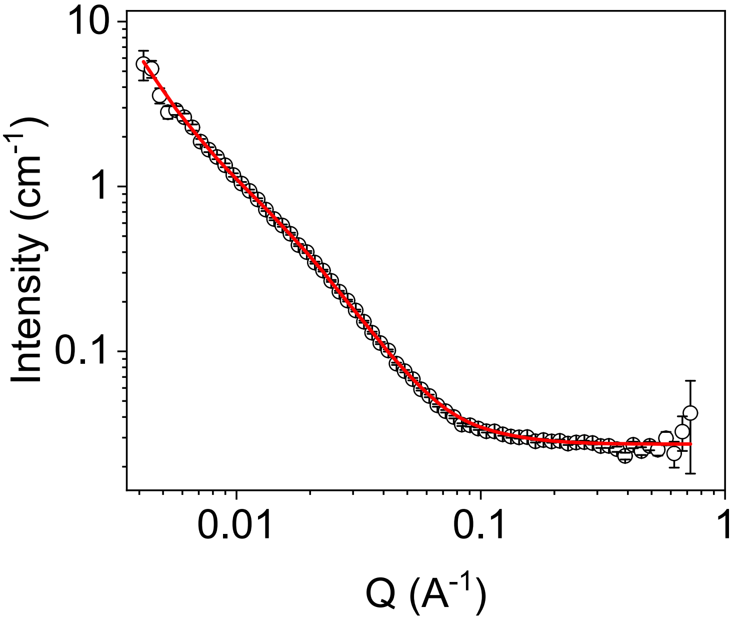

Figure S11. Small angle neutron scattering data for a neutral buffered solution of NDI-GF at pD 6 after one electrochemical cycle and relaxation for several days (black circles) fitted to a flexible elliptical cylinder model combined with power law (red data)

Table S4. Parameters of SANS model fit for NDI-GF *ex situ* before reduction

**NDI-GF *Ex situ* before reduction**	**Flexible elliptical cylinder + power law**
	**Value**
Scale a	4.99E-05
Scale b	4.99E-05
Background (cm^{-1})	0.003755
Length (Å)	762.08
Kuhn length (Å)	60.074
Radius (Å)	19.512
Ratio Axis	1.8167
Power	2.36
Range	0.00416-0.66844
Chi^2	4.8026

Table S5. Parameters of SANS model fit for NDI-GF *ex situ* after reduction, then reoxidised

**NDI-GF *Ex situ* electrochemically reduced and relaxed**	**Flexible elliptical cylinder + power law**
	**Value**
Scale a	1.19E-04
Scale b	3.32E-06
Background (cm^{-1})	0.02576
Length (Å)	1.32E+03
Kuhn length (Å)	45.638
Radius (Å)	7.1657
Ratio Axis	6.2301
Power	2.5443
Range	0.00416-0.72191
Chi^2	1.5126

DISCUSSION ABOUT DATA COLLECTED FROM *IN SITU* MEASUREMENT

The initial Kuhn length is approximately 20 Å lower than that calculated from the fit to the measurement in a standard cuvette, calculated radius is approximately 8 Å larger and the axis ratio approximately 2 units smaller.

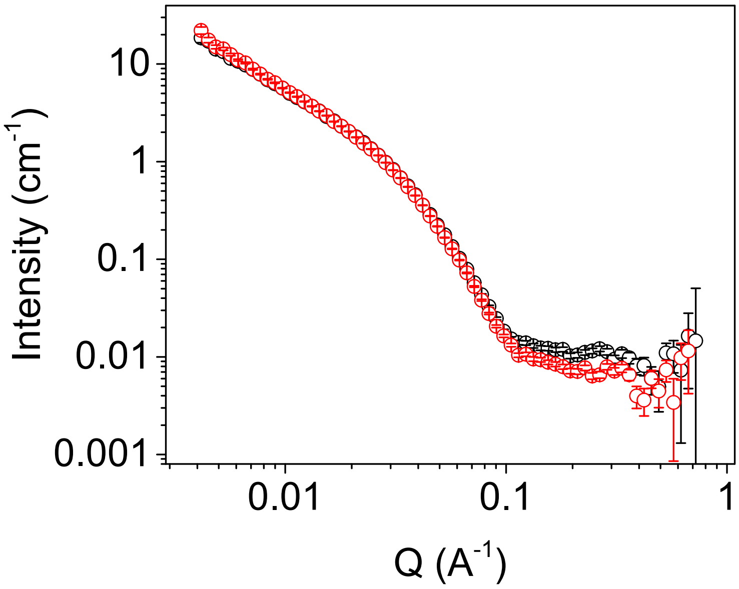

Figure S12. SANS data for a neutral buffered solution of NDI-GF at pD 6 in a spectro-electrochemical cell (black circles) and a standard quartz cuvette (red circles).

Table S6. Parameters of SANS model fit for NDI-GF *ex situ* before reduction

**NDI-GF *in situ* before reduction**	**Flexible elliptical cylinder + power law**
	**Value**
Scale a	6.76E-04
Scale b	3.10E-06
Background (cm^{-1})	0.01023
Length (Å)	1031.50
Kuhn length (Å)	40.947
Radius (Å)	25.132
Ratio Axis	1.9368
Power	2.8273
Range	0.00416-0.57308
Chi^2	4.8588

**NDI-GF *in situ* electrochemically reduced**	**Flexible elliptical cylinder + power law**
	**Value**
Scale a	4.89E-04
Scale b	1.42E-06
Background (cm^{-1})	0.011439
Length (Å)	985.70
Kuhn length (Å)	43.057
Radius (Å)	23.333
Ratio Axis	2.1808
Power	2.9305
Range	0.00416-0.72191
Chi^2	4.0192
**NDI-GF *in situ* electrochemically oxidised**	**Flexible elliptical cylinder + power law**
	**Value**
Scale a	5.32E-04
Scale b	1.73E-06
Background (cm^{-1})	0.014325
Length (Å)	958.25
Kuhn length (Å)	45.445
Radius (Å)	24.908
Ratio Axis	2.0408
Power	2.8958
Range	0.00416-0.72191
Chi^2	4.1256

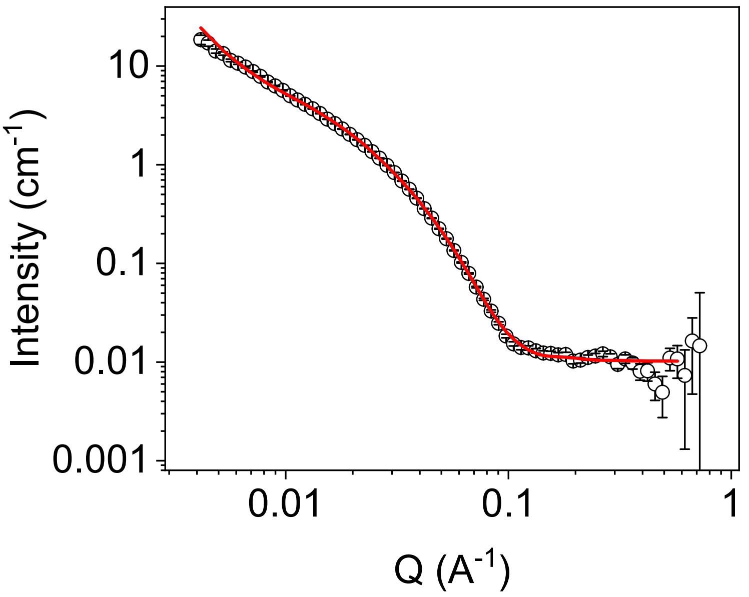

Figure S13. Small angle neutron scattering data for a neutral buffered solution of NDI-GF at pD 6 (black circles) fitted to a flexible elliptical cylinder model combined with power law (red data). Data taken in spectro-electrochemical cell.

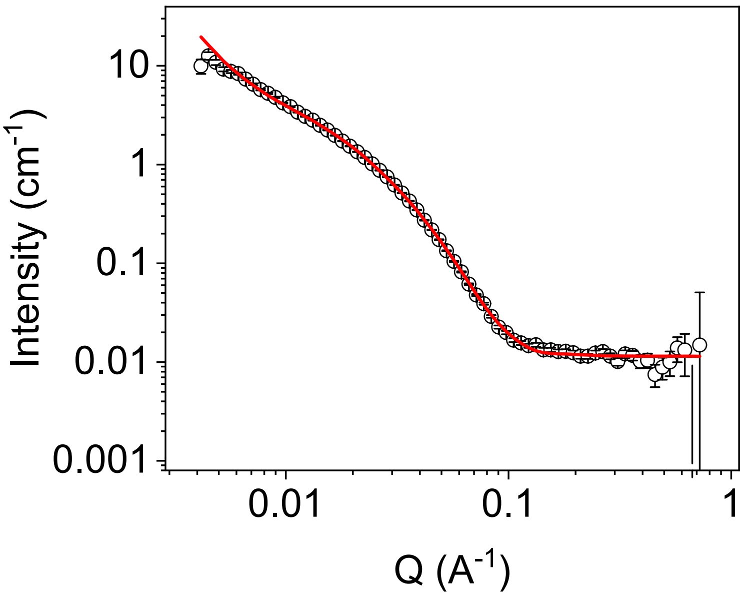

Figure S14. Small angle neutron scattering data for a buffered reduced solution of NDI-GF at pD 6 (black circles) fitted to a flexible elliptical cylinder model combined with power law (red data). Data taken in spectro-electrochemical cell.

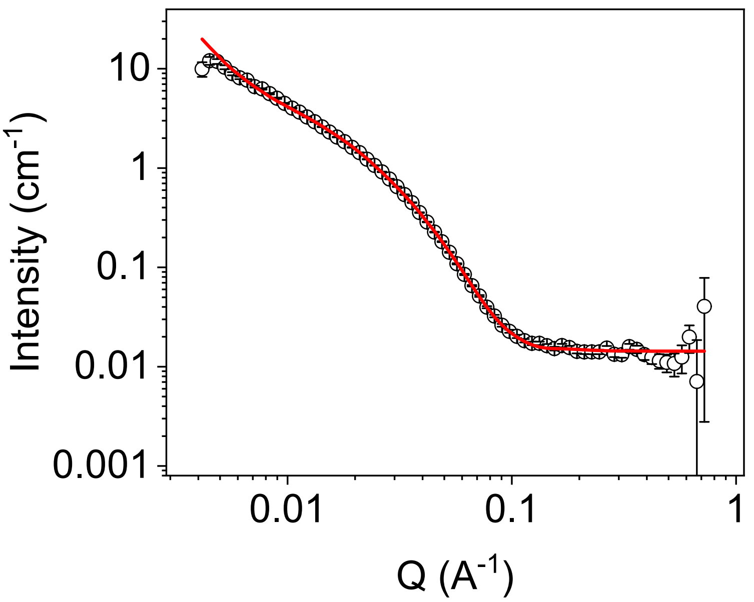

Figure S15. Small angle neutron scattering data for a buffered electrochemically reoxidised solution of NDI-GF at pD 6 (black data) fitted to a flexible elliptical cylinder model combined with power law (red data). Data taken in spectro-electrochemical cell.

4. References

1. Gonzalez, L.; Liu, C.; Dietrich, B.; Su, H.; Sproules, S.; Cui, H.; Honecker, D.; Adams, D. J.; Draper, E. R., Transparent-to-dark photo- and electrochromic gels. *Communications Chemistry* **2018,** *1* (1), 77.
2. Chen, L.; Morris, K.; Laybourn, A.; Elias, D.; Hicks, M. R.; Rodger, A.; Serpell, L.; Adams, D. J., Self-Assembly Mechanism for a Naphthalene−Dipeptide Leading to Hydrogelation. *Langmuir* **2010,** *26* (7), 5232-5242.
3. https://www.palmsens.com/software/ps-trace (accessed 22 Feb).
4. Draper, E. R.; Adams, D. J.; Fuentes-Capparos, A. M.; Schweins, R., New electrochemical approach to monitor gelation self-assembly and kinetics. Institut Laue-Langevin (ILL), 2021.
5. Draper, E. R.; Randle, R. I.; Cavalcanti, L., Investigating the effect of pH and functionalisation upon the self-assembly of NDIs. STFC ISIS Neutron and Muon Source, 2020.
6. https://www.sasview.org (accessed 11/04/2022).
